# Seroprevalence of HIV, hepatitis b, and hepatitis c among opioid drug users on methadone treatment in the netherlands

**DOI:** 10.1186/1477-7517-7-25

**Published:** 2010-10-26

**Authors:** Imke Schreuder, Marianne AB van der Sande, Matty de Wit, Monique Bongaerts, Charles AB Boucher, Esther A Croes, Maaike G van Veen

**Affiliations:** 1 Department of Virology, Erasmus MC, (Dr. Molewaterplein 50), Rotterdam (3000 CA) the Netherlands; 2 Department of Epidemiology and Surveillance, Centre for Infectious Disease Control, National Institute of Public Health and Environment, (Antonie van Leeuwenhoeklaan 9) Bilthoven (3721 MA), the Netherlands; 3 Department of Epidemiology, Documentation and Health Promotion, Public Health Service, (Nieuwe Achtergracht 100) Amsterdam (1018 WT), the Netherlands; 4 Division Addiction Care Group, Mondriaan centre, (Valkenburgerweg 17) Heerlen (6411 BM), the Netherlands; 5 Department of Prevention, Trimbos-institute, (Da Costakade 45) Utrecht (3521 VS), the Netherlands; 6 Julius Centre for Health Sciences and Primary Care, University Medical Centre Utrecht, (Heidelberglaan 100) Utrecht (3508 GA), the Netherlands

## Abstract

**Background:**

Injecting drug users (IDU) remain an important population at risk for blood-borne infections such as human immunodeficiency virus (HIV), hepatitis B virus (HBV) and hepatitis C virus (HCV). In the Netherlands, a program is being implemented to offer annual voluntary screening for these infections to opioid drug users (ODUs) screened in methadone care. At two care sites where the program is now operating, our study aimed to estimate the seroprevalence among ODUs screened for HIV, HBV and HCV; to evaluate HBV vaccination coverage; and to assess the feasibility of monitoring seroprevalence trends by using routine annual screening data.

**Methods:**

Opioid drug users on methadone treatment are routinely offered voluntary screening for infectious diseases such as HIV, HBV and HCV. Data on uptake and outcome of anti-HIV, anti-HBc, and anti-HCV screening among ODUs receiving methadone were obtained from two regions: Amsterdam from 2004 to 2008 and Heerlen from 2003 to 2009.

**Findings:**

Annual screening uptake for HIV, HBV and HCV varied from 34 to 69%, depending on disease and screening site. Of users screened, 2.5% were HIV-positive in Amsterdam and 11% in Heerlen; 26% were HCV-positive in Amsterdam and 61% in Heerlen. Of those screened for HBV, evidence of current or previous infection (anti-HBc) was found among 33% in Amsterdam and 48% in Heerlen. In Amsterdam, 92% were fully vaccinated for HBV versus 45% in Heerlen.

**Conclusion:**

Annual screening for infectious diseases in all ODUs in methadone care is not fully implemented in the Netherlands. On average, more than half of the ODUs in methadone care in Heerlen and Amsterdam were screened for HIV, HBV and HCV. In addition, screening data indicate that HBV vaccination uptake was rather high. While the HIV prevalence among these ODUs was relatively low compared to other drug-using populations, the high HCV prevalence among this group underscores the need to expand annual screening and interventions to monitor HIV, HBV and HCV in the opioid drug-using population.

## Background

Injecting drug users (IDU) and opioid drug users (ODUs) remain at high risk for blood-borne infections with human immunodeficiency virus (HIV), hepatitis B virus (HBV), and particularly hepatitis C virus (HCV) [1-5]. This is due mainly to high transmission risk associated with the sharing of injection equipment and, depending on the virus, to sexual risk behaviour [1-3]. An estimated 25.000 ODUs are currently living in the Netherlands [6-8], of whom approximately 15% inject drugs. About 12,000 ODUs receive outpatient methadone treatment, which is around 50% [7]. This treatment is one of many harm reduction interventions, like syringe exchange programs, which began in Amsterdam in 1984 and spread around the country [9]. Methadone was prescribed on a limited scale to morphine addicts as early as 1968. Methadone distribution programs became more active around 1990, when it became clear that HIV was reaching epidemic levels among drug users in the capital [7,10].

In the Netherlands, harm reduction refers to a range of pragmatic and evidence-based public health policies designed to reduce the harmful consequences of drug use and other high-risk activities [11,12]. Of the opioid drug-using population, roughly 75% regularly use methadone, as opposed to approximately 40% ten years ago [13,14]. Some use methadone on a regular basis, others only occasionally. The methadone programs are primarily intended for harm reduction rather than drug rehabilitation [14]. A total of 11 institutions for care and treatment of drug users control the management of 85 methadone posts countrywide. They are accessible and free for all patients, as methadone is fully covered under the basic health insurance system. While offering methadone, the posts also facilitate education and monitoring of the drug-using population.

In 2005, national guidelines on opiate maintenance treatment were published to support the quality of methadone care [15]. They included a strong recommendation to screen all methadone users annually for such infectious diseases as HIV, HBV and HCV, and to offer treatment to those who test positive. This recommendation is now being gradually implemented at methadone sites across the country. In addition, HBV vaccination is offered to susceptible drug users, including IDUs and ODUs, through the national Hepatitis B Vaccination Campaign [9].

Previous national studies among ODUs and IDUs showed a high burden of HIV, HBV and HCV [13,16-21], although in recent years, the proportion of IDU among newly diagnosed HIV patients has gradually declined in much of the Netherlands, in association with a decline in injecting [10,22]. In 2008, IDU was considered to be the most likely transmission route for 5% of all registered HIV cases in the country [16]. In 1998, 26% of the IDU population in and outside methadone care were HIV-positive in Amsterdam [18] and, in the same year, 22% of the IDU population in Heerlen was HIV-positive [19].

The Netherlands is a low-endemic country with an estimated HBsAg prevalence of 0.3-0.5%, where HBV transmission is restricted mainly to risk groups [23]. The total number of acute HBV patients reported in the Netherlands is an underestimation of the true number of cases, since less than half of infected individuals have symptoms, and not all patients have been reported [23]. There have been few recent studies of HBV among Dutch IDUs [9], but available data indicate the prevalence of markers of previous infection is as high as 35% in The Hague in 2000 [20] and 68% in Heerlen and Maastricht in 1998 [19]. Since the Netherlands has not implemented universal HBV vaccination, it is important to monitor the effect of the National hepatitis B vaccination campaign at risk groups such as drug users.

For HCV, it was estimated that 60,000 people with chronic HCV live in the Netherlands, with only 5,000 to 10,000 of them being aware of their status [4]. Approximately 50 acute HCV cases are reported annually, and more than half are associated with drug use in general [16,17]. The prevalence of anti-HCV varied between 35% in Rotterdam in 2003 [20] and 74% in Heerlen in 1996 [21].

To improve insight into the current burden of infectious diseases among drug users being screened, data can be explored from the annual screening programs now operating at a few methadone posts. We used these data to assess the prevalence among ODUs screened of HIV, HBV and HCV in two different regions, as well as HBV vaccination coverage. We also assessed the utility of using annual screening data to monitor HIV, HBV and HCV prevalence in the opioid drug using population.

## Methods

Data on HIV, HBV and HCV screening of opioid drug users (ODUs) were obtained from methadone posts in Amsterdam and Heerlen, the Netherlands. The Dutch definition for problematic ODU is "injecting drug use or using opioids, cocaine and/or amphetamine on a regular base (min 3/week)" [Methadone treatment centres, personal communication 2010] [8]. As in other regions, methadone treatment is dispensed in various programs by GPs and nurses working from multiple locations and mobile units. These two regions were amongst others of interest to the Ministry of Health, Sports and Welfare, in part because their prevalence of HIV, HBV and HCV among IDUs were relatively high [18,19,21]. They are also of interest due to their established screening programs, which make data available for study and disease monitoring. However, both regions differ greatly in size and their history of drug use.

In Amsterdam, the capital of the Netherlands, methadone has been prescribed since the 1980s [7], providing substitution treatment to a variety of drug users from all over the country and abroad who have come to the capital for drug use. A relatively large proportion of drug users are immigrants from the Caribbean who are less likely to inject drugs than drug users of Dutch background. An estimated 70-80% of Amsterdam's drug users are covered by the low-threshold methadone services across the city [7].

In Heerlen, in the southern part of the Netherlands, many drug users reside in adjacent regions in Germany, Belgium, and France. The proportion of those who inject is quite high. A study conducted in Heerlen by Carsauw et al. in 1997 showed that 69% of the study participants had injected drugs during the previous 6 months [19,21]. The methadone substitution treatment started in Heerlen around 1997 [24].

Amsterdam started voluntary screening for HIV, HBV and HCV at its methadone posts in 2002. For this study, data from the HIV and HBV screening were available from 2006 to 2008. For HCV screening, results of 2004-2008 were obtained. Heerlen has been screened for all these infections since 2003. Information from screening of both regions was used to estimate the seroprevalence among ODUs under methadone treatment for all three infections and to assess HBV vaccination coverage. For each client, only most recent screening results were available and included. In addition, a number of individual details were collected (e.g. gender, date of birth, screening and vaccination coverage, test results, start of treatment for HIV, chronic HBV and chronic HCV). Moreover, data on prevalences for Heerlen reflect a longer period of time than data from Amsterdam, which were only based on 2006-2008. Therefore, these prevalences of both HBV and HIV in Amsterdam might be higher if we take into account the positive cases of the years before 2006. No data is collected on modes of drug use. However, from personal communication we know that approximately 60% in Heerlen and 40% in Amsterdam has ever injected drugs [Methadone treatment centre Heerlen and Amsterdam, personal communication 2010].

Screening is carried out in collaboration with regional laboratories. To estimate the HIV prevalence, we used data from HIV-antibody tests, provided positive results were confirmed. To assess HBV status, we used data from anti-HBc serological tests. Data on HBsAg status were not available. To assess HCV status, results from anti-HCV tests were available. No data on HCV-RNA were available. It should be noted that the antibody tests for HBV and HCV indicate exposure to the virus, but cannot determine if ongoing infections are present.

## Findings

In total, 2566 ODUs were registered in methadone care in Amsterdam between 2004 and 2008. Of these, 2024 were (also) registered between 2006 and 2008. In Heerlen, 287 ODUs were in care from 2003-2008.

### HIV prevalence

A large majority (81%) of HIV-positive ODUs in Amsterdam and Heerlen were male, and by far most (92.5%) were aged above 40 years (Table 1).

**Table 1:** Demographics of drug users found positive for HIV, HBV and HCV in screening at methadone posts in Amsterdam and Heerlen.

	HIV		HBV (anti-HBc)	HCV (anti-HCV)	
	Amsterdam*	Heerlen**	Heerlen**	Amsterdam***	Heerlen**
	**N = 31**	**N = 20**	**N = 93**^**ξ**^	**N = 227**	**N = 115**

Gender:					
- Male	21 (67%)	19 (95%)	65 (70%)	233 (67%)	80 (70%)
- Female	10 (33%)	1 (5%)	28 (30%)	117 (33%)	35 (30%)
Age (years):					
< 30	1 (3%)	0	2 (2%)	5 (1%)	4 (3%)
- 30 - 39	2 (7%)	1 (5%)	7 (8%)	51 (15%)	13 (11%)
- 40 - 49	19 (61%)	14 (70%)	47 (50%)	170 (49%)	65 (57%)
≥ 50	9 (29%)	5 (25%)	37 (40%)	124 (35%)	33 (29%)

* Data of 2006-2008, ** Data of 2003-2008, *** Data of 2004-2008

^ξ ^No data on demographics for HBV in Amsterdam were available

In Amsterdam, 1231/2024 (61%) of the ODUs in care were screened for HIV between 2006 and 2008, and 31/1231 (2.5%) were found positive. In Heerlen, 179/287 (62%) of those in care were screened for HIV between 2003 and 2008, and 20/179 (11%) were found positive (Table 2). Those found HIV-positive in Heerlen were all co-infected with HCV, 65% were anti-HBc positive.

**Table 2:** Seroprevalence of HIV in the two regions.

	Number in methadone care	HIV screening coverage N (%)	HIV prevalence N (%)
Amsterdam*	2024	1231 (61%)	31 (2.5%)
Heerlen**	287	179 (62%)	20 (11%)

* Data of 2006-2008, ** Data of 2003-2008

### HBV prevalence and HBV vaccination uptake

In Amsterdam, 680/2024 (34%) of the ODUs in care were screened for HBV from 2006 to 2008. Of these, 225/680 (33%) had antibodies against HBV (anti-HBc). In total 1469 ODUs were vaccinated against HBV between 2002 and 2008, either full or partially. The estimated vaccination coverage among ODUs in Amsterdam in 2006-2008 was 92%. Completion of HBV vaccination was unknown.

In Heerlen, 197/287 ODUs (69%) were screened for HBV between 2003 and 2008, of whom 93 were anti-HBc positive (48%), mostly male. Of all ODUs in care in Heerlen, 130/287 (45%) persons completed their vaccination course against HBV. Interestingly, of the HIV and HCV-positive individuals, HBV vaccination was completed by 26% and 25%, respectively. Of all ODUs in care who were not vaccinated (n = 157), 22 stated they did not want to get the vaccination; 46 started vaccination but have not yet had their second and/or third vaccine (Table 3).

**Table 3:** Seroprevalence of HBV and vaccination coverage in the two regions.

	Number in methadone care N	HBV screening coverage N (%)	HBV prevalence anti-HBc N (%)	HBV vaccination coverage N (%)
Amsterdam*	2024	680 (34%)	225 (33%)	1469 (92%)***
Heerlen**	287	197 (69%)	93 (48%)	130 (45%)

* Data of 2006-2008, ** Data of 2003-2008, *** Data of 2002-2008

### HCV prevalence

Among the HCV-positive ODUs in Amsterdam and Heerlen, 70% were male and 86.5% were aged 40 years and above (Table 1).

In Amsterdam, 1359/2566 (53%) of the ODUs in care were screened for HCV from 2004 to 2008, and 350/1359 (26%) were positive for HCV antibodies. In 2008, 53/350 (15%) HCV-positive ODUs started treatment. In Heerlen, 190/287 (66%) of ODUs in care were screened for HCV between 2003 and 2008, and 115/190 (61%) were positive. Of these, 55 (48%) have started HCV treatment (Table 4).

**Table 4:** Seroprevalence of HCV in the two regions.

	Number in methadone care	HCV screening N (%)	HCV prevalence N (%)
Amsterdam*	2566	1359 (53%)	350 (26%)
- starting treatment^1^			53 (15%)
Heerlen**	287	190 (66%)	115 (61%)
- starting treatment			55 (48%)

^1^Obtained from the Dutch-C project of the public health centre in Amsterdam within the Amsterdam Cohort Studies among drug users, * Data of 2004-2008, ** Data of 2003-2008

## Discussion

It has been possible to establish routine screening programs for HIV, HBV and HCV among ODUs in methadone care in the Netherlands. Expanding annual screening programs and strengthening coverage will enable improved care for this vulnerable group, and can provide relevant surveillance data to monitor these epidemics among ODUs. Initial results from this screening program show that a significant group, primarily for HBV, do not yet receive such screening. For HBV, this could be affected given that a specific group of drug users, such as IDUs and ODUs, should get vaccinated as part of the national hepatitis B vaccination campaign [23].

Among those screened in two regions, HIV prevalence was relatively low in Amsterdam (2.5%) but higher in Heerlen (11%). Of those screened for HBV, evidence of current or previous infection (anti-HBc) was found among 33% in Amsterdam and 48% in Heerlen HBV vaccination coverage was relatively high in Amsterdam (92%) but only 45% in Heerlen. The prevalence of anti-HCV was higher than HIV, ranging from 26% in Amsterdam to 61% in Heerlen.

In the past, studies among drug users in and outside methadone treatment in Amsterdam have demonstrated HIV prevalences higher than our finding [18]. Previous cross-sectional surveys among IDU in Heerlen found HIV prevalences of 16.3% in 1996 and 21.6% in 1998. These prevalences are also higher compared to our finding of 11% [19-21], however these studies were restricted to IDUs only whereas our study focused on ODUs, including those injecting drugs. Behavioural surveys have shown that injecting drugs has decreased and is now less popular [10,22], which could explain part of these differences. In addition, in comparison to cross-sectional studies, testing in a treatment setting has been performed selectively for those not already known to be HIV-infected. Finally, the population of ODUs who still inject is aging, and many HIV-infected drug users have died in the last decade, which can also result in lower HIV prevalence.

Although a direct comparison with previous studies is not possible, the higher HIV prevalence found by other studies may reflect another drug user's population that is recruited outside methadone treatment settings. These users may have a higher burden of HIV than those in care. Moreover, studies have shown that methadone treatment is associated with a lower risk of HIV infection, probably by discouraging injecting and encouraging better knowledge of risk factors [25,26].

The current HIV prevalence among ODUs in our study is comparable to trends of other western European countries. However, in Eastern Europe and outside of Europe, HIV rates have increased in recent years [27] and suggest an increasing incidence of HIV infection among people who inject drugs [28]. Alertness on possible re-emergence of HIV among drug users in the Netherlands is therefore essential to prevent relapse.

In 2000, more than half of the persons in a methadone clinic population in America had evidence of HBV exposure [29]. In this study, the proportion of persons who ever injected drugs was 78.7%. Our data, indicating both past and acute infections, shows comparable results. In the UK, the overall seroprevalence of exposure markers for HBV (anti-HBc) was 48% among ODUs in and outside the methadone setting [30]. However, this study was conducted many years earlier.

For the current study, only data from anti-HBc serological tests were available to assess HBV prevalence among those who are screened, unfortunately no data on HBsAg status were available. In case of a positive anti-HBc test, it is recommended to also assess the HBsAg status to identify and consequently interrupt the risk for individual transmission, as well as transmission on population level.

Our study showed a reasonably high number of ODUs completing their vaccine course for HBV, however, vaccination must still be increased further for Heerlen [30,31]. Besides protecting against HBV, it may help drug users to develop a stronger pro-health attitude, leading to less HCV-related risk behaviour, according to Quaglio et al [31]. By November 2009, the national Hepatitis B Vaccination Campaign in the Netherlands had estimated vaccination coverage of approximately 15,000 drug users, of whom approximately 60% completed their three-part vaccination within 6 months [23,32].

The prevalence of HCV in this study is lower than found by international studies conducted in comparable methadone settings from 1999 to 2004. Those studies show an overall prevalence of 67-96% [5,29,33-36] and even higher prevalence among drug users who inject drugs (around 95%). Our results are compatible with studies conducted outside methadone settings in 2006-2007, which found prevalences of 40-70% in samples from Bulgaria, Georgia, Germany, France, Italy, Poland, and Ukraine, with prevalence of 80-90% in Germany, France, Italy, Poland, Romania, and Spain [4,27]. Moreover, the proportion of HCV-positive individuals starting HCV treatment is fairly high in our study, particularly in Heerlen, compared to studies that show proportions of 6%, 9%, and 35% [37-39].

The variation in the proportion starting HCV treatment between the two regions (48% vs. 15%) might be explained by several factors. First, starting HCV treatment is a time consuming process and requires much personal capacity. The absolute number of ODUs screened for HCV in methadone care in Amsterdam is 7 times higher compared to Heerlen, however, the actual number of persons who started HCV treatment is comparable in both centres. Secondly, 95 HCV positive (and HIV negative) persons in Amsterdam are currently in anticipation of a new, and probably more effective, drugs to start HCV treatment [Methadone treatment centre, personal communication 2010].

The most common contributors to the relatively low levels of treatment rates for HCV are the strict criteria to start treatment, which are similar between the two centres [Methadone treatment centre Amsterdam and Heerlen, personal communication 2010], insufficient knowledge among drug users, unwillingness to face side effects (e.g. depression), lack of initial evaluation and adherence to additional appointments [37-39]. It is therefore of highly importance to improve the understanding of HCV status and HCV transmission among drug users.

Possible explanations for the persistently higher prevalence of HIV, HBV and HCV in Heerlen compared to Amsterdam could be the ongoing higher level of injecting drug use and related risk behaviour (e.g. borrowing of syringes) combined with the influx of HIV-positive drug users from adjacent regions and countries [21]. National drug monitoring in the Netherlands has found injecting drug use more popular in the southern region than in others (19% vs. 10%) [39]. Moreover, we have presented the HIV and HBV prevalence for Heerlen that reflect a longer period than data from Amsterdam, which were only based on 2006-2008. The prevalence of both HBV and HIV in Amsterdam might be higher if we also take into account the positive cases of the years before 2006.

Our study results should be interpreted in the context of a number of limitations. The implementation of screening for infectious diseases has been conducted differently in the two study regions, perhaps creating differences between their data. In addition, differences might seem exaggerated because data were missing from enough surrounding regions to provide context. Besides this, it would be of interest to also collect data on risk factors such as routes of administration of drug use, needle sharing and sexual risk behaviour. Based on the available data collected in Heerlen en Amsterdam, however, this was not possible. We therefore recommend collecting such data in the voluntary infectious disease screening.

Another limitation is that we only targeted ODUs in our study, whereas other subgroups of drug users may be at risk of infectious diseases as well. However, in the Netherlands, the injection of drugs has decreased substantially in the last years [6-8] and injection of crack is rare. Moreover, most IDUs are included in opioid substitution programs.

Finally, the availability of data on HBV and HCV-antibody tests only made it not possible to distinguish whether infections have been cleared or remain active. Following infection, less than 5% of HBV infected adults develop chronic HBV infection, regardless if a person injects drugs [40]. Twenty-five to fifty percent of IDUs develop acute hepatitis C [1]. IDUs with a chronic HBV infection and acute HCV infections are the groups in need of medical evaluation and the groups to target to interrupt ongoing transmission.

In conclusion, annual screening for infectious diseases of ODUs in methadone care is not fully implemented in the Netherlands. However, two regions with such implementation have generated data for assessing the prevalence of infectious diseases. Although collecting data should be improved to use screening results for monitoring trends, they show a relatively low HIV and HBV prevalence among ODUs screened, but it is evident that the HCV prevalence is high. We therefore recommend enhancing the implementation of voluntary infectious disease screening in all methadone treatment settings nationwide. Drug users who are diagnosed positive can be provided with early treatment, which will benefit them while also reducing further transmission. Furthermore, since many ODUs are not in methadone care, it is of importance to raise awareness about HCV and facilitate its early diagnosis among incarcerated drug users and others outside the methadone setting. Harm reduction interventions and early detection of new HIV, HBV, and HCV infections are of vital importance to provide adequate treatment which can interrupt ongoing transmission and lead to a general gain in health benefit.

## List of abbreviations

EMCDDA: European Monitoring Centre for Drugs and Drug Addiction; HBV: Hepatitis B Virus; HCV: Hepatitis C Virus; HIV: Human Immunodeficiency Virus; IDU: Injecting Drug User; IDUS: injecting drug users; ODU: opioid drug use; ODUS: opioid drug users; RIOB: Richtlijn Opiaat Onderhoud Behandeling; RIVM: Rijksinstituut voor Volksgezondheid en Milieu (national institute for public health and the environment).

## Competing interests

The authors declare that they have no competing interests.

## Authors' contributions

IS and MvV carried out the study; have made substantial contributions to conception and design, acquisition of data, and analysis and interpretation of data; and contributed to the manuscript. MvdS and CB have been involved in drafting the manuscript or revising it critically for important intellectual content. MB, MdW and EC all provided information on the infectious diseases screening and have given final approval of the version to be published.

All authors have read and approved the final manuscript for publication.
